# Improved Survival Prediction by Combining Radiological Imaging and S-100B Levels Into a Multivariate Model in Metastatic Melanoma Patients Treated With Immune Checkpoint Inhibition

**DOI:** 10.3389/fonc.2022.830627

**Published:** 2022-04-14

**Authors:** Simon Burgermeister, Hubert S. Gabryś, Lucas Basler, Sabrina A. Hogan, Matea Pavic, Marta Bogowicz, Julia M. Martínez Gómez, Diem Vuong, Stephanie Tanadini-Lang, Robert Foerster, Martin W. Huellner, Reinhard Dummer, Mitchell P. Levesque, Matthias Guckenberger

**Affiliations:** ^1^ Department of Radiation Oncology, University Hospital Zurich and University of Zurich, Zurich, Switzerland; ^2^ Department of Dermatology, University Hospital Zurich and University of Zurich, Zurich, Switzerland; ^3^ Department of Nuclear Medicine, University Hospital Zurich and University of Zurich, Zurich, Switzerland

**Keywords:** melanoma, immunotherapy, survival analysis, combined models, outcome modeling, tumor burden, S-100B, LDH (Lactate dehydrogenase)

## Abstract

**Purpose:**

We explored imaging and blood bio-markers for survival prediction in a cohort of patients with metastatic melanoma treated with immune checkpoint inhibition.

**Materials and Methods:**

94 consecutive metastatic melanoma patients treated with immune checkpoint inhibition were included into this study. PET/CT imaging was available at baseline (Tp0), 3 months (Tp1) and 6 months (Tp2) after start of immunotherapy. Radiological response at Tp2 was evaluated using iRECIST. Total tumor burden (TB) at each time-point was measured and relative change of TB compared to baseline was calculated. LDH, CRP and S-100B were also analyzed. Cox proportional hazards model and logistic regression were used for survival analysis.

**Results:**

iRECIST at Tp2 was significantly associated with overall survival (OS) with C-index=0.68. TB at baseline was not associated with OS, whereas TB at Tp1 and Tp2 provided similar predictive power with C-index of 0.67 and 0.71, respectively. Appearance of new metastatic lesions during follow-up was an independent prognostic factor (C-index=0.73). Elevated LDH and S-100B ratios at Tp2 were significantly associated with worse OS: C-index=0.73 for LDH and 0.73 for S-100B. Correlation of LDH with TB was weak (r=0.34). A multivariate model including TB change, S-100B, and appearance of new lesions showed the best predictive performance with C-index=0.83.

**Conclusion:**

Our analysis shows only a weak correlation between LDH and TB. Additionally, baseline TB was not a prognostic factor in our cohort. A multivariate model combining early blood and imaging biomarkers achieved the best predictive power with regard to survival, outperforming iRECIST.

## Introduction

Since the approval of ipilimumab in 2011, the first therapeutic agent targeting the CTLA-4 immune checkpoints pathway, immune checkpoint inhibitors have revolutionized treatment of metastatic melanoma patients (1–3). In 2014, the PD-1 inhibitors nivolumab and pembrolizumab were approved by the FDA, extending treatment options. These treatments have improved long-term survival and remission rates of melanoma patients. Nevertheless, many patients do not benefit from immune checkpoint inhibition and severe, potentially life-threatening complications have been reported (4). This highlights the urgent need for early treatment response assessment and prediction in order to limit potentially serious side effects and to redirect patients toward alternative treatment options as early as possible.

Previous work on predictive biomarkers mainly focused on features extracted from blood and tumor biopsy (5–8). Biopsy results from tumor samples have shown encouraging results, but remain limited both in time and anatomical coverage due to the intrinsic invasiveness of biopsies. Imaging and blood-derived parameters are therefore considered as promising biomarker candidates (9) because repetitive and longitudinal assessment can be performed during follow-up as well as imaging-based tumor heterogeneity investigation by assessment of individual metastatic lesions and analysis of heterogeneity within individual lesions.

For imaging-based response assessment, RECIST 1.1 (Response Evaluation Criteria in Solid Tumors) is widely used and validated (10). iRECIST has been developed to account for response patterns specific to immuno-oncology (11, 12). However, response assessment using iRECIST and RECIST 1.1 is based on selected target lesions, and therefore does not represent a comprehensive analysis of the total tumor burden and metastatic pattern. Additionally, specific lesions contributing to a patient’s tumor burden are categorized as “non measurable”. These include infiltrative lesions, unclearly delimited lesions, bone lesions without significant soft tissue component and lesions treated with radiotherapy (13) - all of which are common in metastatic disease.

Several studies have investigated prognostic potential of imaging and blood biomarkers (5, 14) as well as correlations among them. LDH has long been assumed to reflect tumor burden or tumor invasive potential (15, 16), but a clear *in-vivo* connection is still to be established.

Although both imaging and serum biomarkers are regularly measured during clinical follow-up, the prognostic value of combined blood and imaging data in metastatic melanoma has not been investigated. In our study, we aimed to explore the relationship between blood biomarkers in melanoma, such as lactate dehydrogenase (LDH), C-reactive protein (CRP) and S-100B, with imaging-based total tumor burden in metastatic melanoma patients treated with immune checkpoint inhibition. We also investigated the prognostic value of imaging-based tumor burden and blood biomarkers for survival prediction. Finally, we aimed to develop a multivariate survival model combining imaging and blood markers.

## Materials and Methods

### Patient Cohort

In this retrospective single-institution study, we included 94 consecutive metastatic melanoma patients, who were treated with either single checkpoint inhibition (aPD-1) or double checkpoint inhibition (aPD-1 and aCTLA-4) at the University Hospital of Zurich between 2013 and 2019. The following exclusion criteria were applied: patients with only small metastases at baseline (<1 cc), lack of follow-up/baseline imaging, patients with no positron emission tomography with 2-deoxy-2-[fluorine-18]fluoro-D-glucose integrated with computed tomography (18F-FDG PET-CT) at baseline, patients with brain metastases only, patients treated with immune checkpoint inhibitors (ICI) as adjuvant therapy.

### Endpoints

The primary endpoint of this study was patient overall survival after treatment onset. Early response to immunotherapy was evaluated using dedicated 18F-FDG-PET/CT within 3 months before treatment start (Tp0) and either 18F-FDG-PET/CT or contrast-enhanced CT at 3 months ± 2 months (Tp1) and 6 months ± 2 months (Tp2). Patient response to ICI was evaluated using iRECIST tumor response criteria between Tp0 and Tp2. For the purpose of our study, we grouped progressive disease (iPD) and unconfirmed progressive disease (iUPD) status at Tp2 as “progressive”, whereas complete response (iCR), partial response (iPR) and stable disease (iSD) were considered as “non-progressive”.

### Imaging and Lesion Segmentation

Whole-body (head to feet; if the primary tumor was located in the lower extremities) or partial-body (head to upper thighs) 18F-FDG-PET/CT served as baseline imaging in all patients. Follow-up imaging (Tp1 and Tp2) was either 18F-FDG-PET/CT or contrast-enhanced CT of the chest, neck and abdomen. All metastatic lesions were manually segmented by a single observer and checked by a second one. Segmentation was done using MIM software based on assessment reports provided by the nuclear medicine and radiology departments.

### Imaging Markers

Lesions were categorized according to their anatomic locations. Volume, longest and shortest diameter were calculated for each metastasis at each timepoint. Total TB was calculated using the sum of longest diameters. Changes in TB were computed using change in longest diameters and change in overall volume. Immunotherapy specific response evaluation criteria in solid tumors (iRECIST) were applied based on Tp0 and Tp2 and served as baseline in this study. For univariate biomarkers analysis, we explored volume- and diameter-based tumor burden, but as both showed similar results and because the latter is more widely used in clinical practice and easier to compute, we focused on it in the multivariate analysis.

### Blood Markers

Blood parameters were obtained by routine hematology measurements in the Department of Hematology of University Hospital Zurich according to standard diagnostics procedures. Analysis of blood markers consisted of CRP, LDH and S-100B serum levels measured during follow-up. The measurements closest to the respective imaging dates were used for modeling and for correlation analysis of blood markers and TB. The median measurements from a month-wide window were used to evaluate differences in blood marker levels between progressing and non-progressing patients (according to iRECIST) at consecutive months after the start of the treatment. This last point also allows corrections for outliers.

### Statistical Analysis

The Cox proportional hazards model was used for survival analysis. Performance of the univariate models was evaluated with hazard ratio (HR) and the concordance index (C-index), confidence intervals were calculated using bootstrapping. We also evaluated a multivariate Cox proportional hazard model using exhaustive search for every pair and triplet of marker combination. For this model, performance was evaluated with the C-index using 100-times repeated 5-fold cross-validation. Survival models and parameters were also evaluated using area under curve (AUC) after logistic regression at 24 months. For this evaluation, features were directly extracted from the longitudinal models. Distribution of blood marker levels between progressive and non-progressive patients were compared using Mann-Whitney *U* test.

### Software

The analysis was done in the Python programming language (version 3.7.10). For numerical statistical analysis, we used NumPy & SciPy (17) and scikit-learn (18). Survival models were computed with Lifelines (19). Family-wise error rate was computed with statsmodels (20). For visualization, we used the Matplotlib (21) and Seaborn (22) libraries.

## Results

### Patient Characteristics

Detailed patient characteristics is provided in **Table 1**. Our cohort consisted of 94 patients and 598 lesions. There were 66 men and 28 women. The median patient age was 67.5 years. Patients had a median value of five metastatic lesions at baseline and tumor burden of 10.64 cm (sum of lesion diameters) and 38.80 cm^3^ (sum of lesion volumes). 77 patients were treated with aPD-1, while 17 patients were treated with a combination of aCTLA-4 and aPD-1. Patients with higher tumor burden were more often treated with combination treatment (median TB: 8.32 cm vs. 13.97 cm; p = 0.04).

**Table 1:** Patient and treatment characteristics.

Patients demographics	
Total patients	94
Age [years]	
Median	67.5
Q1-Q3	53–74
Range	33–93
Sex	
Female	28
Male	66
**Metastatic lesions at baseline**	
Total number of lesions	598
Median	5
Q1-Q3	2–9
Range	1–47
Sum of lesion diameters at baseline [cm]	
Median	10.64
Q1-Q3	5.26-25.52
Range	1.44–92.21
Sum of lesion volumes at baseline [cm^3^]	
Median	38.80
Q1-Q3	7.90-90.09
Range	1.01–602.05
**AJCC pathological prognostic stages at baseline**	
IIIa	1
IIIc	5
IIId	1
IV	87
**Treatment information**	
aPD1*	77
2 mg/kg pembrolizumab	64
3 mg/kg nivolumab	8
aPD1 + aCTLA:	17
1 mg/kg nivolumab + 3 mg/kg ipilimumab	16
2 mg/kg pembrolizumab + 1 mg/kg ipilimumab	1
**Disease evolution at Tp2 based on iRECIST**	
iCR	7
iPR	30
iSD	42
iPD & iUPD	15

*Dosage data was not available for five patients.

Metastases at baseline were most frequently located in lymph nodes (29%), followed by lungs (21%) and liver (18%). More detailed information about metastatic lesion locations at all three time points are provided in **Table 2**.

**Table 2:** Metastases locations among all patients during follow-up. “Other” include: retroperitoneal, adrenal gland, heart, throat, orbits and lesions with ambiguous anatomical localisation.

Site	Tp0	Tp1	Tp2
lymph node	177 (29%)	116 (28%)	113 (29%)
lung	125 (21%)	54 (13%)	44 (11%)
liver	109 (18%)	98 (24%)	89 (23%)
bone	53 (9%)	33 (8%)	27 (7%)
intraperitoneal	35 (6%)	20 (5%)	27 (7%)
subcutaneous	26 (4%)	31 (7%)	22 (6%)
muscle	17 (3%)	17 (4%)	23 (6%)
spleen	10 (2%)	8 (2%)	6 (2%)
other	49 (8%)	37 (9%)	33 (9%)
TOTAL	601 (100%)	414 (100%)	384 (100%)

### Patient Response and Survival

**Figure 1:**
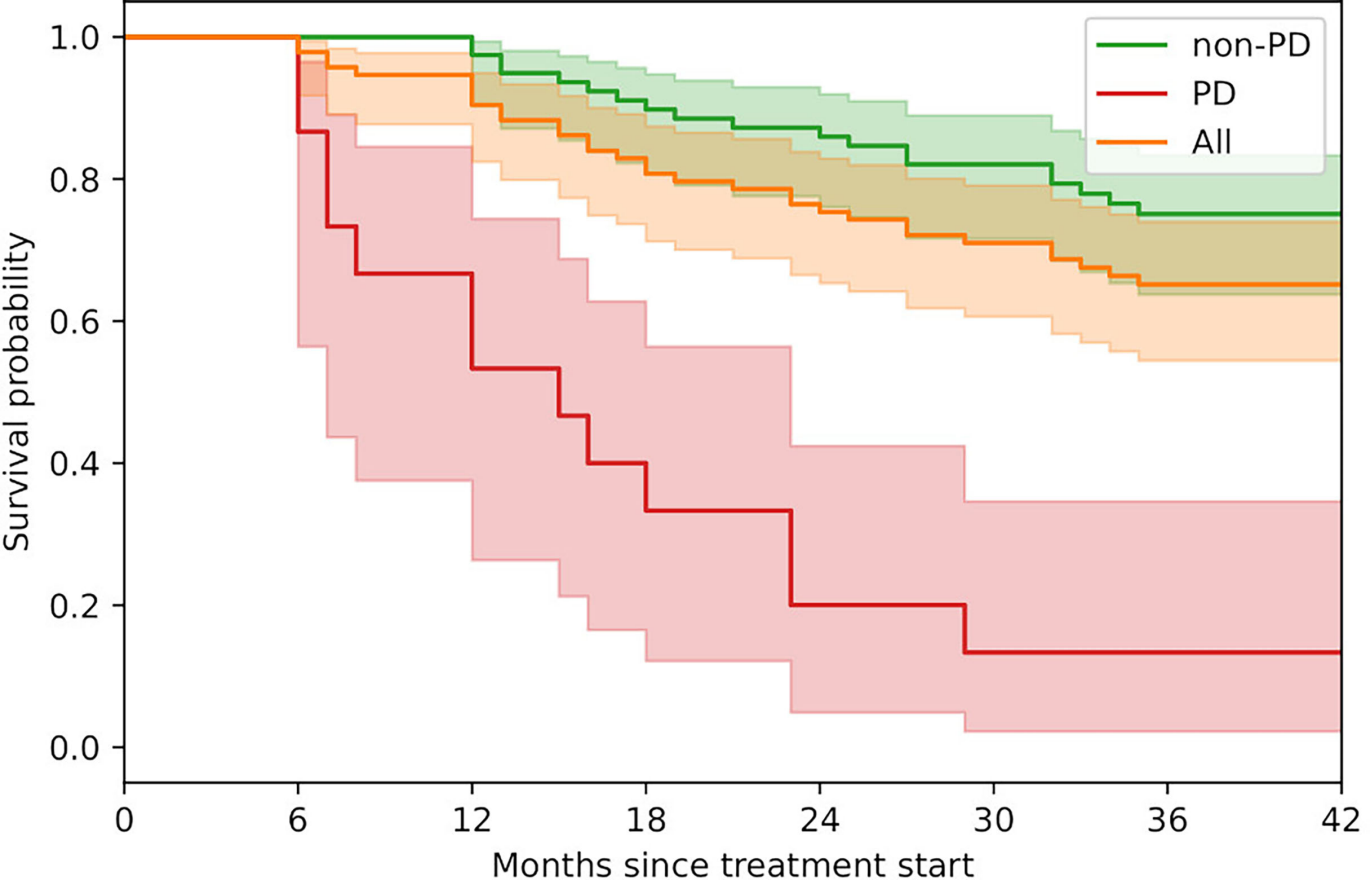
Kaplan-Meier survival curves. Progressive patients (PD; n=15) include iPD and iUPD. Non-progressive patients (non-PD; n=79) include iCR, iPR and iSD. Shaded areas represent the 95% confidence intervals.

The majority of patients (84%) responded to the therapy and showed either significant decrease in tumor burden (iCR (n=7) or iPR (n=30)) or stable tumor burden with no new lesions during 6 months follow-up (iSD; n=42). The remaining 16% of patients showed an increase in tumor burden exceeding 20% (iUPD and iPD; n=15). Overall survival for the full patient cohort at 2 and 3 years was 76% and 56%, respectively. The Kaplan-Meier survival curves are shown in **Figure 1**. The difference in overall patient survival between single and double checkpoint inhibition was not statistically significant (p = 0.31).

### Relationships Between Blood and Imaging Markers

**Figure 2:**
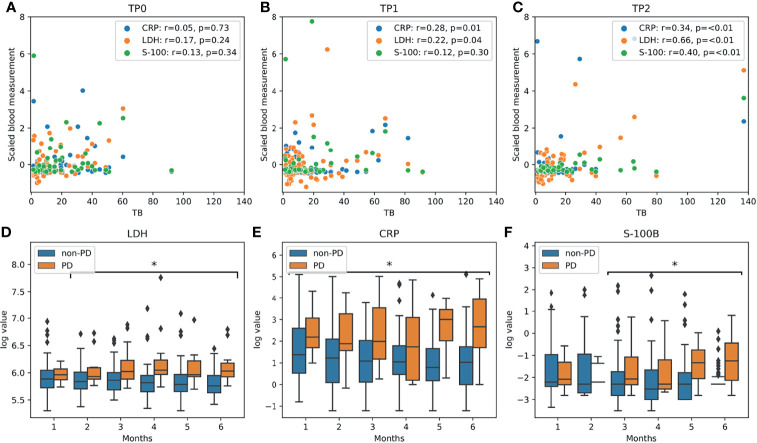
Blood marker analysis: **(A–C)** scatter plots of tumor burden and blood markers for all three timepoints. Pearson’s *r* coefficients are given in the legend box. **(D–F)** Differences in LDH, CRP and S-100B levels during follow-up between progressive (PD: iPD and iUPD; n=15) and non-progressive patients (non-PD: iCR, iPR and iSD; n=79). Statistically significant different levels (p<0.05, using Mann-Whitney *U* test) for monthly follow-up are highlighted with an asterisk (*).

The relationship between absolute blood levels of LDH, CRP and S-100B levels and absolute TB was weak (**Figures 2A–C**). Correlations were strongest at Tp2 (average *r* = 0.51) and weakest at Tp0 (average *r* = 0.15). The analysis of blood marker levels between progressive and non-progressive patients revealed a temporal pattern (**Figures 2D–F**). There was no significant difference in LDH and S-100B levels for the first one month after baseline. However, with longer follow-up, progressive patients were characterized by significantly (p < 0.05) increased serum levels of LDH starting at month 2, and S-100B at month 3 compared to non-progressive patients. CRP levels were significantly elevated among progressive patients, starting after the first month of follow-up.

### Predictive Power of Individual Imaging and Blood Markers

There was no significant association between overall survival and patient age, sex or the ICI type. Blood marker levels at baseline were not associated with overall survival. High tumor burden at baseline also did not correlate with high mortality (tp=0.72; **Table 3**). Progressive disease (iPD and iUCP) at Tp2 was strongly associated with higher mortality, with HR=9.11 (4.50–18.44) and C-index=0.68 (0.60–0.75). However, other biomarkers, such as TB at Tp1 and Tp2, TB change at Tp1 and Tp2, LDH, S-100B and CRP levels at Tp2, showed even higher association with overall survival (**Tables 3**, **4**). Appearance of new metastatic lesions during follow-up (Tp0–Tp2) was also a predictive factor with HR=7.00 (3.57–13.74) and C-index=0.73 (0.66–0.80).

**Table 3:** Results from univariate Cox proportional hazard regression on imaging biomarkers with hazard ratio (HR), C-index, AUC at 24 months and *p*-values. Parameters statistically significant at FWER=0.05 are in bold.

Predictor	HR	C-index	AUC 24 months	*p*
Tp0 sum of diameter	1.00 (0.99–1.02)	0.56 (0.46–0.66)	0.56 ± 0.15	0.7191
**Tp1 sum of diameter**	**1.03 (1.01–1.04)**	**0.67 (0.57–0.76)**	**0.69** ± **0.13**	**0.0003**
**Tp2 sum of diameter**	**1.03 (1.02–1.04)**	**0.71 (0.61–0.81)**	**0.74** ± **0.12**	**<0.0001**
Tp0 sum of volume	1.00 (1.00–1.01)	0.56 (0.46–0.65)	0.62 ± 0.13	0.0815
Tp1 sum of volume	1.00 (1.00–1.00)	0.69 (0.60–0.77)	0.72 ± 0.13	0.0123
**Tp2 sum of volume**	**1.00 (1.00–1.00)**	**0.72 (0.63–0.81)**	**0.76** ± **0.12**	**<0.0001**
**Appearance of new lesions**	**7.00 (3.57–13.74)**	**0.73 (0.66–0.80)**	**0.73** ± **0.1**	**<0.0001**
**Number of new lesions**	**1.10 (1.06–1.15)**	**0.76 (0.68–0.83)**	**0.75** ± **0.1**	**<0.0001**
Tp0-Tp1 ratio diameter	1.20 (1.03–1.41)	0.68 (0.59–0.77)	0.69 ± 0.12	0.0216
**Tp0-Tp2 ratio diameter**	**1.27 (1.14–1.41)**	**0.73 (0.63–0.82)**	**0.75** ± **0.12**	**<0.0001**
**Tp1-Tp2 ratio diameter**	**1.54 (1.24–1.91)**	**0.67 (0.56–0.77)**	**0.70** ± **0.12**	**0.0001**
Tp0-Tp1 ratio volume	1.00 (0.98–1.01)	0.27 (0.18–0.37)	0.34 ± 0.21	0.6363
**Tp0-Tp2 ratio volume**	**1.44 (1.29–1.6)**	**0.74 (0.64–0.83)**	**0.76** ± **0.13**	**<0.0001**
Tp1-Tp2 ratio volume	1.32 (1.09–1.61)	0.71 (0.6–0.82)	0.74 ± 0.12	0.0055
**iRECIST**	**9.11 (4.50–18.44)**	**0.68 (0.60–0.75)**	**0.69** ± **0.09**	**<0.0001**

**Table 4:** Results from univariate Cox proportional hazard regression on blood biomarkers with hazard ratio (HR), C-index, AUC at 24 months and *p*-values. Parameters statistically significant at FWER=0.05 are in bold.

Predictor	HR	C-index	AUC 24 months	*p*
Tp0 CRP	1.01 (0.94–1.08)	0.52 (0.41–0.63)	0.45 ± 0.12	0.7622
Tp0 LDH	1.46 (0.65–3.32)	0.51 (0.41–0.61)	0.48 ± 0.13	0.3618
Tp0 S-100B	0.94 (0.85–1.05)	0.57 (0.47–0.67)	0.57 ± 0.13	0.2866
Tp1 CRP	1.15 (1.02–1.3)	0.59 (0.48–0.7)	0.62 ± 0.13	0.0201
**Tp1 LDH**	**4.69 (2.24–9.84)**	**0.64 (0.54–0.73)**	**0.62** ± **0.13**	**<0.0001**
Tp1 S-100B	1.05 (1.01–1.09)	0.66 (0.56–0.76)	0.66 ± 0.13	0.0219
Tp2 CRP	1.06 (1.01–1.1)	0.71 (0.62–0.8)	0.72 ± 0.12	0.0106
**Tp2 LDH**	**11.79 (5.28–26.31)**	**0.73 (0.62–0.82)**	**0.74** ± **0.12**	**<0.0001**
**Tp2 S-100B**	**1.06 (1.03–1.1)**	**0.73 (0.64–0.82)**	**0.75** ± **0.13**	**0.0004**

### Multivariate Model of Patient Survival

Using blood markers only, the best performing model had a mean C-index = 0.75 ± 0.10, similar to tumor burden only model (mean C-index = 0.75 ± 0.10) and appearance of new metastatic lesions (mean C-index = 0.76 ± 0.09). Combining parameters significantly increased the predictive power of our survival models with the best models combining appearance of new lesions and S-100B levels (**Table 5**).

**Table 5:** Best performing multivariate Cox models after 100x-repeated 5-fold cross-validation. Models are categorized according to parameters used: blood, tumor burden, and appearance of new lesions as well as combinations of them. iRECIST was included for comparison. Models showing statistically significant (p < 0.05) improved performance compared to iRECIST are in bold.

Model parameters	C-index	AUC 24 months	Parameter categories
Tp2 CRP Tp2 S-100B	0.75 ± 0.10	0.75 ± 0.12	Blood
Tp0-Tp1 ratio diameter Tp1-Tp2 ratio diameter	0.75 ± 0.10	0.77 ± 0.12	Tumor burden
Number of new lesions	0.80 ± 0.08	0.75 ± 0.10	New lesions
Appearance of new lesions Tp2 S-100B	**0.83** ± **0.07**	**0.84** ± **0.09**	New lesions Blood
Appearance of new lesions Tp1-Tp2 ratio diameter	**0.83** ± **0.07**	**0.84** ± **0.09**	New lesions Tumor burden
Tp0 S-100B Tp1 S-100B Tp0-Tp2 ratio diameter	0.76 ± 0.10	0.75 ± 0.12	Blood Tumor burden
Appearance of new lesions Tp1 S-100B Tp1-Tp2 ratio diameter	**0.83** ± **0.07**	**0.83** ± **0.10**	New Lesions Blood Tumor burden
iRECIST	0.68 ± 0.08	0.68 ± 0.09	iRECIST

## Discussion

Results of this retrospective study based on a single-center cohort of metastatic melanoma patients treated with either single or double immune checkpoint inhibition show that OS is not always associated with baseline TB nor with baseline blood levels of LDH, S-100B or CRP; potentially widening treatments accessibility to patients currently excluded based on those baseline markers levels. Additionally, our results contradict the hypothesis that LDH at baseline is a surrogate marker for the patient’s tumor burden. A prediction model combining absolute S-100B blood levels during early follow-up with imaging based response criteria achieved accurate and significantly improved OS prediction compared to the established standard of iRECIST.

TB at baseline showed no significant association with survival in our cohort, with tumor burden defined as either sum of longest diameters of all lesions or sum of volumes of all lesions. This is in contrast to earlier work, showing significantly increased mortality among patients with higher TB at baseline (23, 24). Our study, however, specifically excludes patients treated with ICI as adjuvant to surgery. Such patients usually have low TB and better survival rates, potentially explaining the observed difference. However, the observed response dynamic among such patients is vastly different to what is seen among patients who are not candidates for curative surgery, excluding them is therefore meaningful. Another potential factor contributing to this discrepancy between our results and previous studies is the lack of an established gold standard for tumor burden assessment. Indeed, while previous works mainly focus on RECIST 1.1 (5, 24), such an analysis underestimates tumor burden in cases where multiple lesions are present in the same organ. Based on our results, patients with high tumor burden at baseline should not be excluded from ICI protocols or clinical trials because of concerns of a worse prognosis.

Studies on predictive or prognostic biomarkers in melanoma have shown promising results, some of which are now integrated into staging protocols (25). Nevertheless, response evaluation criteria remain strictly focused on imaging findings, while other biomarkers are usually assumed to be linked to metastatic tumor burden. This is the case of serum LDH level, often considered a proxy for tumor burden in standard clinical practice (15). However, several studies, including ours, have failed to demonstrate a close correlation between serum LDH level and tumor burden (23, 26, 27). Our study shows, however, that LDH remains a strong predictor of mortality as early as three months after initiation of ICI therapy, independently of TB. On the other hand, elevated LDH and CRP levels are often seen in many different health-related events, some of which are associated with ICI adverse events, so it is also possible that the increased hazard ratio is linked to these events (28, 29). Nonetheless, the fact that LDH levels collected during follow-up in our study were predictive of iRECIST status at 3 and 6 months indicates that this biomarker remains relevant for metastatic disease assessment. Other groups have reported similar results (28).

Another plasma biomarker investigated in our study, CRP, also showed significant association with progressive outcome and survival. CRP, an acute-phase protein produced by the liver in response to elevated cytotoxin levels, has already been suggested as a potential biomarker for several cancers including melanoma (30, 31). The relationship between CRP and cancer has been hypothesized to exist either through chronic inflammation, CRP playing a causal role or by the elevated levels reflecting an underlying malignant or premalignant state. In our cohort, CRP was a poor prognostic factor for survival at baseline and showed a weak correlation with TB. However, this biomarker showed stronger predictive power for patient survival later during follow-up, with significantly higher levels observed in progressive patients. These results could suggest an underlying inflammatory state, unrelated to overall TB, causing both elevated serum levels of the protein and progression during treatment course. In such cases, patients could benefit from combined blood and imaging investigation to improve diagnostic and prognostic accuracy.

Compared to the abundantly distributed and unspecific circulating protein CRP and cytosolic enzyme LDH, the cytoplasmic protein S-100B is considered as a more specific and reliable marker for malignant melanoma (32). Additionally, elevated serum S-100B has been shown to be associated with reduced survival (28). In our study, S-100B was inferior to LDH as an individual predictor of survival, but showed the largest added value in multivariate models when combined with imaging biomarkers. S-100B is more specific to cells originally derived from the neural crest, which includes melanocytes. Almost all melanomas strongly express S-100B. Its role in melanoma, although not completely understood, is thought to be linked to its interaction with p53 and activation of STK38/NDR1 (33, 34) and therefore interfering with cell proliferation and programmed death. More importantly and as opposed to CRP and LDH, S-100B is not known to be associated with adverse events seen with ICI therapy.

The multivariate survival analysis showed similar results between a TB-based model and blood marker levels at Tp2. However, the appearance of new lesions proved to be a crucial parameter in both imaging-only and combined models, outperforming models based solely on TB or blood markers. We did not see any benefit in including blood markers to a model based on new lesion appearance and TB change or adding TB to models based on lesion appearance and blood markers. Interestingly, a model based on TB and new lesion appearance performed significantly better than iRECIST. This finding suggests that total TB carries more significance than its surrogate based only on “target lesions”. However, total TB estimates can be resource-intensive, especially in patients with heavy tumor load, and can be subject to inter- and intraobserver delineation variability. On the other hand, circulating S-100B levels are easily available and inexpensive to process. Finally, a model based on a combination of S-100B levels at Tp2 and appearance of new lesions performed similarly to the best models using changes in TB and significantly better than iRECIST.

Both serum biomarkers and imaging findings are routinely investigated in metastatic melanoma patients, but as they are the responsibility of different specialties, the combined predictive power of these markers is seldom investigated. Our analysis indicates that such a multivariate and multidisciplinary approach may have the potential to improve diagnostic and prognostic performance while reducing workload.

The main limitation of our study is the lack of external validation and a retrospective design with a relatively small number of patients treated in a single institution. To address these issues, we used repeated cross-validation, bootstrapping and multiple testing correction to validate our results internally. This allowed us to generate reliable model performance scores and reduce risk of overfitting. Manual delineation of lesions is another limitation as it affects reproducibility (35). However since no automated tools are at the moment available for whole body assessment of extension, it is still the most widely used one. Another limitation was exclusion of lesions smaller than 1 cc. This was dictated by limitations of the imaging resolution and uncertainty of metastatic disease state at baseline. Additionally, blood sampling in our cohort was performed at irregular intervals, because the combination of imaging and blood marker response evaluation is seldom used in clinical practice. To limit the impact of this irregular sampling, we used median monthly values for each patient. Future investigations would have to be conducted with a dedicated cohort combining blood and imaging data in order to comprehensively investigate the relationship between both modalities.

In conclusion, the combination of image-based and blood-based biomarkers outperformed iRECIST or blood-only models for the prediction of patient survival in metastatic melanoma treated with immune checkpoint inhibition. Furthermore, although LDH is often assumed to be a proxy for TB, our analysis showed only a weak correlation between LDH and TB. This finding highlights the importance of considering LDH levels and image-based TB as complementary factors in survival analysis of melanoma patients. Furthermore, prognostic evaluation and patient selection based on baseline TB seems to be unreliable and could withhold treatment from patients who may still benefit from it.

## Data Availability Statement

The raw data supporting the conclusions of this article will be made available by the authors, without undue reservation.

## Ethics Statement

The studies involving human participants were reviewed and approved by Kantonale Ethikkommission Zürich approval nr. 2019-01012. The patients/participants provided their written informed consent to participate in this study.

## Author Contributions

Conception and design: SB, HG, STL, MG, ML. Development of methodology: SB, HG, STL, MG, ML. Acquisition of data: SB, HG, LB, SH, JMG, MP, RD. Analysis and interpretation of data: SB, HG, STL, MG. Writing, review, and revision of the manuscript: SB, HG, LB, SH, MP, MB, JMG, DV, STL, RF, MH, RD, ML, MG. Study supervision: RD, ML, MG. All authors contributed to the article and approved the submitted version.

## Funding

This work was supported by Cancer Research Center, Comprehensive Center Zurich, University Hospital Zurich (CRC_13, to LB); SwissNational Fund (SNF 310030_170159, to HG); and European Training Network MELGEN funded consortium (no. 641458, to SH).

## Conflict of Interest

MH received a grant from the CRPP AI Oncological Imaging Network of the University of Zurich, grants from GE Healthcare and a fund by the Alfred and Annemarie von Sick legacy for translational and clinical cardiac and oncological research.

RD has intermittent, project focused consulting and/or advisory relationships with Novartis, Merck Sharp & Dhome (MSD), Bristol-Myers Squibb (BMS), Roche, Amgen, Takeda, Pierre Fabre, Sun Pharma, Sanofi, Catalym, Second Genome, Regeneron, Alligator, T3 Pharma, MaxiVAX SA, Pfizer and touchIME outside the submitted work.

The remaining authors declare that the research was conducted in the absence of any commercial or financial relationships that could be construed as a potential conflict of interest.

## Publisher’s Note

All claims expressed in this article are solely those of the authors and do not necessarily represent those of their affiliated organizations, or those of the publisher, the editors and the reviewers. Any product that may be evaluated in this article, or claim that may be made by its manufacturer, is not guaranteed or endorsed by the publisher.
